# Clinicopathologic features of colorectal carcinoma: features predicting higher T-stage and nodal metastasis

**DOI:** 10.1186/s13104-018-3183-2

**Published:** 2018-01-19

**Authors:** Atif Ali Hashmi, Shumaila Kanwal Hashmi, Navaira Ali, Komal Thara, Rabia Ali, Muhammad Muzzammil Edhi, Naveen Faridi, Amir Khan

**Affiliations:** 1 Department of Histopathology, Liaquat National Hospital and Medical College, Karachi, Pakistan; 2 Department of Pathology, CMH Institute of Medical Sciences, Multan, Pakistan; 3 Department of Oncology, Liaquat National Hospital and Medical College, Karachi, Pakistan; 4 Department of Surgery, Rhode Island Hospital and Brown University, Providence, RI USA; 5 Department of Medicine, Kandahar University, Kandahar, Afghanistan

**Keywords:** Colorectal carcinoma, Pakistan, Microsatellite instability, Chromosomal instability

## Abstract

**Objectives:**

A rising frequency of colorectal carcinoma has been noted in recent years in Pakistan. In the present study, we aimed to evaluate clinicopathologic features of colorectal carcinoma in our population so that protocols could be developed to stratify patients that may require further biomarker/molecular testing. Furthermore, histological features which predict higher T and N stage were also evaluated.

**Results:**

Median age at diagnosis was 54.5 (19–85) years. 79% cases were of conventional adenocarcinoma while 13% cases were of mucinous carcinoma. Most of the cases were at T3 stage (81%), while 27 and 68% of cases revealed lymphovascular invasion and nodal metastasis respectively. Mucinous and signet ring tumors were associated with a higher N stage. Pre-existing polyp was associated with lower T and N stage. We found a high proportion of our cases to present at advanced T-stage. Tumor grade and lymphovascular invasion were found to be associated with higher N-stage while tumor infiltrating lymphocytes was associated with lower T and N-stage. Moreover, a high frequency of mucinous differentiation may be linked to microsatellite instability in our cases of colorectal carcinoma; therefore, we suggest that microsatellite instability testing in colorectal carcinoma should be evaluated in our setup.

## Introduction

Colorectal carcinoma (CRC) is the third most common malignancy worldwide [1]. A rising incidence of colorectal carcinoma has been noted in recent years, high percentage of which was found to be right sided [2]. There are different pathways of colorectal carcinogenesis including chromosomal instability, microsatellite instability (MSI) and CpG island methylation with overlap between these pathways. Chromosomal instability occurs in about 85% of patients with sporadic CRC and familial adenomatous polyposis and is characterized by aneuploidy, chromosomal rearrangements and accumulations of mutations in oncogenes and tumor suppressor genes [3]. On the other hand, CRC secondary to MSI are because of one of the three possible pathways; germline mutations, sporadic mutations and epigenetic silencing [4, 5]. Germline mutations are associated with Hereditary Non-Polyposis Colon Cancer/Lynch syndrome [6]. The two major pathways of colorectal carcinogenesis are associated with distinct clinicopathologic features. Chromosomal instability pathway CRC are associated with left side (distal) location, older age and pre-existing polyps while MSI pathway CRC are associated with right side (proximal) location and younger age [4]. Studies have shown that MSI associated CRC have specific histologic features. MSI-H tumors are more likely to be multiple, to show polypoid growth pattern, exhibit sharply circumscribed and pushing margins and marked necrosis. Furthermore, MSI-H tumors are more likely to show mucinous or signet ring features as well as microglandular differentiation [7, 8]. Prognosis as well as management strategies differ in these two groups of CRC; therefore immunohistochemical and molecular testing are now routinely recommended for those patients meeting the clinical and histologic criteria [9–11]. Unfortunately in this part of the world these molecular markers are not routinely performed due to limited resources. On the other hand, surrogate clinico-pathologic features are also not widely studied. Therefore we aimed to evaluate clinicopathologic features of CRC in our population so that protocols could be developed to stratify patients requiring further biomarkers in order to be characterized into one of these two groups. Furthermore, histological features which predict higher T and N stage were also evaluated.

## Main text

### Patients and methods

Total 100 patients with CRC were included in the study that underwent primary colonic resection at Liaquat national hospital during 2013 till 2015. Patients with distant metastasis or those that received pre-operative chemo-radiation were excluded from the study. An approval from institutional ethical review committee was taken antecedent to conducting the study. Informed written content was taken from all patients at the time of surgery. After resection, specimens were sent to histopathology laboratory. After gross examination, sections were stained by hematoxylin and eosin method. Cases were examined by two senior histopathologists with more than 5 years of reporting gastrointestinal pathology. Tumor typing and grading was done according to WHO guidelines. Various histologic features were determined according to College of American pathologist guidelines using following criteria.

#### Mucinous histology

Extracellular mucin accumulation bounded either by neoplastic epithelium or stroma. Tumors were subgrouped as mucinous histology being absent, < 10, 10–50 and > 50% of tumor area involved [12].

#### Signet ring cells

Presence of tumor cells with intracytoplasmic mucin and peripherally displaced cresent shaped nucleus, whether present within extracellular mucin pools or infiltrating stroma.

#### Medullary differentiation

Sheets, trabeculae or nests of small to medium sized tumor cells exhibiting syncytial pattern, frequent mitosis and abundant stromal lymphocytic infiltration.

#### Necrosis

Presence of dirty necrosis; further sub-grouped into focal and widespread.

#### Peri-tumoral lymhocytic response

Pronounced lymphoid reaction to tumor composed of lymphoid follicles with germinal centers at tumor edges, not associated with either mucosa or pre-existing lymph node. Two or more large lymphoid aggregates in a section were required for the presence of this feature [13].

#### Intratumoral lymphocytic infiltrate

The presence of small round lymphocytes within neoplastic epithelial cells. This category was subgrouped into mild to moderate (up to 3 intra-epithelial lymphocytes/HPF) and marked (> 3/HPF).

T and N stage was evaluated according to AJCC guidelines as follows,

**Primary tumor (T)**

TX: Primary tumor cannot be assessed
T0: No evidence of primary tumor
Tis: Carcinoma in situ: intraepithelial or invasion of lamina propria
T1: Tumor invades submucosa
T2: Tumor invades muscularis propria
T3: Tumor invades through the muscularis propria into pericolorectal tissues
T4a: Tumor penetrates to the surface of the visceral peritoneum
T4b: Tumor directly invades or is adherent to other organs or structures

**Regional lymph nodes (N)**

NX: Regional lymph nodes cannot be assessed
N0: No regional lymph node metastasis
N1: Metastasis in 1–3 regional lymph nodes
N1a: Metastasis in one regional lymph node
N1b: Metastasis in 2–3 regional lymph nodes
N1c: Tumor deposit(s) in the subserosa, mesentery, or nonperitonealized pericolic or perirectal tissues without regional nodal metastasis
N2: Metastasis in 4 or more regional lymph nodes
N2a: Metastasis in 4–6 regional lymph nodes
N2b: Metastasis in 7 or more regional lymph nodes

### Statistical analysis

Statistical package for social sciences (SPSS 21) was used for data compilation and analysis. Median and standard deviation were calculated for quantitative variables. Frequency and percentage were calculated for qualitative variables. Chi square was applied to determine association. *P* value ≤ 0.05 was considered significant.

### Results

#### Descriptive statistics

Median age at diagnosis was 54.5 (19–85) years with a male to female ratio of 1:1. 70% cases were left sided (sigmoid and rectum). 79% cases were of conventional adenocarcinoma histology while 13% cases were of mucinous carcinoma and 74% cases were moderately differentiated. Most of the cases were at T3 stage (81%) while 27 and 68% of cases revealed lymphovascular invasion and nodal metastasis respectively. Among tumor characteristics 32 and 16% of tumors showed mucinous and signet ring features and medullary features were noted in two cases. Pre-existing polyp was seen in 10% of cases. Necrosis was present in most cases (94%). Marked host response i.e. intra-tumoral and peri-tumoral lymphocytic response was noted in 18 and 15% cases respectively (Table 1).

**Table 1:** Clinicopathological and prognostic features of colorectal carcinoma in studied population

Variable	n (frequency)
Age (years)	
Median	54.5 (19–85)
< 50	37 (37%)
> 50	63 (63%)
Gender	
Male	51 (51%)
Female	49 (49%)
Laterality	
Right	30 (30%)
Left	70 (70%)
Lymphovascular invasion	
Present	27 (27%)
Absent	73 (73%)
T stage	
T1	1 (1%)
T2	7 (7%)
T3	81 (81%)
T4	11 (11%)
N stage	
N0	32 (32%)
N1	30 (30%)
N2a	19 (19%)
N2b	19 (19%)
Tumor grade	
Well differentiated	3 (3%)
Moderately differentiated	74 (74%)
Poorly differentiated	23 (23%)
Tumor type	
Adenoarcinoma, NOS	79 (79%)
Mucinous	13 (13%)
Medullary	2 (2%)
Signet ring	5 (5%)
Perinodal spread	
Present	47 (47%)
Absent	53 (53%)
Mucinous histology (%)	
Absent	68 (68%)
< 10	12 (12%)
10–50	7 (7%)
> 50	13 (13%)
Signet ring differentiation	
Present	16 (16%)
Absent	84 (84%)
Medullary differentiation	
Present	2 (2%)
Absent	98 (98%)
Necrosis	
Absent	6 (6%)
Focal	70 (70%)
Widespread	24 (24%)
Tumor infiltrating lymphocytes	
None	57 (57%)
Mild to moderate	25 (25%)
Marked	18 (18%)
Peri-tumoral lymphocytic response	
None	67 (67%)
Mild to moderate	18 (18%)
Marked	15 (15%)
Pre-existing polyp	
Present	10 (10%)
Absent	90 (90%)

#### Association of clinicopathological and prognostic parameters with T stage and N stage

Association of various clinical and histologic features with prognostic parameters i.e. T and N stage was evaluated. Significant association of tumor features was noted with T and N stage. Poorly differentiated tumors were found to be associated with higher T and N stage. Mucinous and signet ring tumors were associated with a higher N stage. Lymphovascular invasion was found to be associated with nodal metastasis; and perinodal spread with higher T stage. Pre-existing polyp was associated with lower T and N stage. Similarly marked intra-tumoral response was negatively associated with both T and N stage (Tables 2, 3).

**Table 2:** Association of clinicopathological and prognostic parameters of colorectal carcinoma with T stage

Variable	T stage				P value
	T 1	T2	T3	T4	
Age (years)					
< 50	0	2	31	4	0.84
> 50	1	5	50	7	
Gender					
Male	0	2	46	3	0.111
Female	1	5	35	8	
Laterality					
Right	0	2	23	5	0.62
Left	1	5	58	6	
Lymphovascular invasion					
Present	0	0	24	3	0.356
Absent	1	7	57	8	
Grade					
Well differentiated	0	2	1	0	< 0.001*
Moderately differentiated	1	5	65	5	
Poorly differentiated	0	0	15	6	
Tumor type					
Adenocarcinoma, NOS	1	7	64	8	0.65
Mucinous carcinoma	0	0	12	1	
Medullary carcinoma	0	0	2	0	
Signet ring cell carcinoma	0	0	3	2	
Perinodal spread					
Present	0	0	40	7	0.037*
Absent	1	7	41	4	
Mucinous histology (%)					
Absent	1	6	54	7	0.778
< 10	0	0	9	3	
10–50	0	0	7	0	
> 50	0	1	11	1	
Signet ring differentiation					
Present	0	1	13	2	0.97
Absent	1	6	68	9	
Medullary differentiation					
Present	0	0	1	0	0.971
Absent	1	7	80	11	
Necrosis					
Absent	0	0	4	2	0.228
Focal	0	6	56	1	
Widespread	1	1	21	8	
Tumor infiltrating lymphocytes					
None	0	3	50	5	0.047*
Mild-moderate	1	0	19	4	
Marked	0	4	12	2	
Peri-tumoral lymphocytes					
None	0	7	53	7	0.166
Mild-moderate	1	0	14	3	
Marked	0	0	14	1	
Pre-existing polyp					
Present	1	2	7	0	0.004*
Absent	0	5	74	11	

Chi square test applied

* P-value is significant

**Table 3:** Association of clinicopathological and prognostic parameters of colorectal carcinoma with N stage

Variable	N stage				P value
	N0	N1	N2a	N2b	
Age (years)					
< 50	10	8	9	10	0.19
> 50	22	22	10	9	
Gender					
Male	18	13	10	10	0.777
Female	14	17	9	9	
Laterality					
Right	6	11	5	8	0.26
Left	26	19	14	11	
Lymphovascular invasion					
Present	0	8	9	10	< 0.001*
Absent	32	22	10	9	
Grade					
Well differentiated	3	0	0	0	< 0.001*
Moderately differentiated	27	26	16	7	
Poorly differentiated	2	4	3	12	
Tumor type					
Adenocarcinoma, NOS	29	27	16	8	0.006*
Mucinous carcinoma	2	2	2	7	
Medullary carcinoma	1	0	0	1	
Signet ring cell carcinoma	0	1	1	3	
Perinodal spread					
Present	0	13	15	19	< 0.001*
Absent	32	17	4	0	
Mucinous histology (%)					
Absent	26	20	15	7	0.006*
< 10	2	6	2	2	
10–50	3	0	0	4	
> 50	1	4	2	6	
Signet ring differentiation					
Present	1	5	2	8	0.003*
Absent	31	25	17	11	
Medullary differentiation					
Present	1	0	0	0	0.543
Absent	31	30	19	19	
Necrosis					
Absent	1	2	3	0	0.097
Focal	22	20	10	18	
Widespread	9	8	6	1	
Tumor infiltrating lymphocytes					
None	14	15	13	16	0.019*
Mild-moderate	7	9	5	3	
Marked	11	6	1	0	
Peri-tumoral lymphocytes					
None	21	24	12	10	0.639
Mild-moderate	6	3	4	5	
Marked	5	3	3	4	
Pre-existing polyp					
Present	7	2	1	0	0.047*
Absent	25	28	18	19	

Chi square test applied

* P-value is significant

### Discussion

In the current study we reported detailed histopathologic features of colorectal carcinoma in Pakistani patients and features which are associated with higher T-stage and nodal metastasis. Pathologic examination of surgically resected specimens is a key factor influencing further management and includes histologic type, status of margins, pathologic staging and determining various prognostic parameters like lymphovascular invasion, perineural invasion and features of MSI. We found a significantly higher proportion of CRC at higher T-stage (T3/T4) i.e. 92%; which contrasts to early detection of CRC in many regions of the world. This may be due to lack of screening colonoscopy, delayed patient presentation or underlying genetic status of the tumors.

A significant proportion of tumors in our study showed mucinous differentiation i.e. 32% out of which 13% showed more than 50% mucinous component and thus classified as mucinous carcinoma. Mucinous carcinoma usually behaves in a more aggressive fashion compared to conventional adenocarcinoma. On the other hand mucinous differentiation when associated with MSI behaves in a less aggressive way [14, 15]. Mucinous differentiation in our study was associated with a higher frequency of nodal metastasis. Further studies are needed to establish its association with MSI.

Tumor infiltrating lymphocytes (TIL) and peri-tumoral lymphocytic response are markers of MSI and represents activated T-cell cytotoxic immune response in CRC [16]. TIL have been found to be independently associated with improved survival in CRC after curative surgery [17]. 18% of our cases were found to have marked TIL and TIL in our study was found to be associated with lower T and N stage. Moreover, only 10% of cases showed pre-existing polyp which is a marker of chromosomal instability pathway. These findings necessitate the testing of MSI status in CRC cases in our population should be evaluated, especially those tumors which show TIL and mucinous differentiation.

Among other prognostic markers of CRC; tumor grade, poor differentiation and lymphovascular invasion were found to be associated with higher risk of nodal metastasis [18–20]. Our findings are concordant with the literature. We found that higher tumor grade to be significantly associated with higher T-stage. Similarly, tumor grade and lymphovascular invasion were also found to be significantly associated with advanced N-stage.

## Limitations

The main limitation of this study was molecular testing was not done and follow-up of patients was not available. However, the data revealed major clinical implications; we found a high proportion of our cases to present at advanced T-stage. Tumor grade and lymphovascular invasion were found to be associated with higher N-stage while tumor infiltrating lymphocytes was associated with lower T and N-stage. Moreover, a high frequency of mucinous differentiation and TIL may be linked to MSI in our cases of CRC; therefore we suggest that MSI status in CRC should be evaluated in our setup.
